# Medical Opinion and Movement

**Published:** 1909-10-30

**Authors:** 


### Medical Opinion and Movement.

In an article in the *Presse Médicale* Dr. Marcou advocates Auto-serotherapy for inducing the re-absorption of pleural effusions. The method was first tried by Dr. Gilbert, of Geneva, and consists in injecting subcutaneously two cubic cm. of the pleuritic fluid every week. It may be employed in cases of serous or hæmorrhagic effusions, but not purulent. In the majority of cases it is stated that absorption rapidly ensues. Pleuritic effusions in patients with advanced tuberculous lesions are said, however, to be refractory to the treatment. The best results are obtained in cases of extensive effusions in which the treatment is applied early. A glass syringe of two cubic centimetres capacity, with a long needle, is all that is required. The needle is inserted so as to penetrate into the effusion, and the syringe is filled with the pleuritic fluid, the needle is then withdrawn till the point is felt just under the skin. The syringe is turned obliquely and the contents injected subcutaneously, and the needle is then withdrawn. The patient is kept quiet for half an hour or so afterwards, but no dressing is necessary. The author has never observed any local reaction, and the operation is attended with little or no pain. A second injection should be made at the end of a week. For the last four years he has only had to evacuate the fluid once in eighty-four cases of pleural effusion, and all his patients have been cured.

---

The inconveniences and discomfort attending Subcutaneous and Intramuscular Injections have led some authorities to administer tuberculin by the mouth, and good results from this method have been reported. In view of this, Pfeiffer and Persch have carried out some experiments to determine the effect of the digestive ferments upon the active principle of tuberculin, and an account of their work has appeared in the *Wiener Klinische Wochenschrift*. They find that tuberculin submitted to the action of pepsin and of trypsin rapidly loses its activity, and generally becomes completely inactive. Twenty patients who gave a positive cuti-reaction with normal tuberculin were then tested with tuberculin treated with pancreatin. Forty-eight times the reaction was negative and twice it was feebly positive. Supposing then that normal intestinal mucous membrane absorbs the tuberculin, it appears that it would be rendered quite inactive by the digestive ferments, and would be quite inefficacious. The authors suggest that in cases in which general symptoms have been observed to follow the administration of tuberculin by the mouth there may have been a deficiency of digestive ferments, or lesions of the mucous membrane may have facilitated absorption of tuberculin before it was rendered inactive. Experimenting with erepsine from the intestinal mucous membrane of cats, the authors find that the activity of tuberculin is modified but not destroyed to the same extent as by the other ferments, and they think that this points to the active principle of tuberculin being of the nature of an albumose rather than of a peptone.

In the *Archiv für Klinische Chirurgie* appears an account of the work of Lexer, of Königsberg, on Joint Transplantation. His experiments have been limited to the knee-joint, which he has attempted to replace when ankylosed from various causes. When possible he makes a large skin flap so that the skin sutures do not overlie the transplanted joint. The Esmarch bandage is avoided, as any hæmorrhage overlooked between the transplanted and original bone may spoil the result. The bones are divided at right angles to their long axis, and all normal periosteum and tendons are carefully avoided in the excision of the joint. For transplantation material Lexer uses joints from cases of amputation for dry arteriosclerotic gangrene and for trauma. The joint to be transplanted must, of course, be prepared under the most rigid aseptic conditions, and all tissue removed down to the periosteum. One of the most difficult points in the procedure is to secure a satisfactory synovial membrane for the new joint. Hydrocele sac has been transplanted in two cases, but with no very satisfactory result. In other cases synovial membrane has been secondarily implanted some time after the primary graft of the joint. All forms of nails or sutures are to be avoided in fixing the transplanted joint, as they lead to the formation of granulation-tissue and to bone absorption. A further trouble is the arrangement of muscles for the proper function of the joint, as the muscles may be atrophied from disuse and disease. No definite rules can be laid down; any of the muscles near the joint may have to be utilised in the way most suited to the case. When the *x*-rays show that bony union has taken place, passive and then active movements may be commenced in the joint.

---

In a contribution to *La Semaine Médicale*, Dr. Katzenstein, of Berlin, proposes a new method of Surgical Intervention for Varicose Veins by which he seeks to enclose the affected vein within the subjacent muscular tissue. He points out that the usual methods of operation—ligature of the saphenous vein or subcutaneous excision of the varicose veins—are not altogether satisfactory. They may fail in effecting a cure, and there is always the possible danger of pulmonary embolism in consequence of the induced thrombosis. The author bases his method upon physiological considerations. He argues that one of the chief propelling factors by which the blood returns to the heart from the lower limbs is the contraction of the muscles. The saphenous and other superficial veins are especially liable to become varicose, because the muscles work upon them only on one side, and they are only covered on the other side by fascia, which readily yields to the dilating pressure of the blood. Varicose veins are much more common in women from lack of exercise, resulting in deficient muscular tone. It is therefore with this view in mind that Dr. Katzenstein proposes to enclose the veins within the muscular tissues of the thigh, and so effect a physiological cure, the contractions of the muscles thus helping to the fullest extent the return flow of the blood and supporting the walls of the veins. In carrying out the operation, an incision is made over the varicose vein, which is isolated from the surrounding tissue. The aponeurosis is then incised and the muscular tissue divided in the direction of the fibres and the vein buried within it. The muscle is then closed again over the vein by a continuous catgut suture, and the aponeurosis and skin are then similarly sewn up. The operation has so far been carried out in six cases with satisfactory results. The author thinks that hæmorrhoids might be similarly treated by enclosing the affected veins within the fibres of the sphincter ani.

TALALGIA, a painful affection of the heel which exists without apparent lesion, is, according to Schwartz (*Progrès Médicale*), of two varieties. In the first, which is the more common, talalgia is accompanied by an old-standing subcalcanean exostosis, the result of long-continued injury, and is consequently common in waiters, shop-assistants, and others whose occupation necessitates much walking and standing. The subjacent soft parts are irritated and a chronic hygroma is formed, and in some cases the fibro-fatty tissue is chronically inflamed with a consequent neuritis of the neighbouring nerve filaments. Any of these lesions causes pain, the primary cause of which is the exostosis. The second variety exists without exostosis. A hygroma of the subcalcanean bursa is the usual cause of pain in these cases. This may result from trauma, but is more commonly of gonorrhœal origin. Before proceeding to treat talalgia, the heel should be radiographed to determine the condition of the bony parts. A horseshoe-shaped incision is then made in the heel down to bone, the cutaneous and fatty layers being next turned forwards and examined. If the bursa or the fatty layers are found to be inflamed they are removed. Attention is afterwards directed to the bony parts, and if present the bony exostosis is removed. The muscles in front of it are divided in such a way as to free the point of this excrescence, which is then cut away entire along with its periosteum by means of scissors. In this way a permanent good result can be ensured.

WINKELMANN, in the *Deutsche Zeitschrift für Chirurgie*, calls attention to the fact that it is possible to open the Peritoneal Cavity without cutting a muscle and almost bloodlessly, by pushing aside the rectus muscle for almost its entire width and throughout its entire length after splitting its anterior sheath, and then incising the posterior sheath and peritoneum transversely. The preliminary skin incision is usually made transversely or obliquely, but in some cases a longitudinal incision may be preferable. The anterior and posterior sheath of the rectus are cut transversely. After operation the abdomen can be closed in such a way as to offer greater assurance against hernia than can be secured by any other method. The author has employed this method with most gratifying success in numerous cases, including operations for appendicitis, cholelithiasis, diseases of the stomach and affections of the uterus and its appendages.

IN a recent number of the *Medical Sentinel*, Bettman remarks on the pathogenesis and treatment of pyorrhœa alveolaris. Since the disease is essentially an ulcerative process, all irritants must be removed, and to this end the teeth must be carefully scaled, if necessary after the application of a local anæsthetic to the gums. Loose teeth must be fixed either by metal splints or with silk ligatures. Each pocket must be then injected with a few drops of lactic or trichloracetic acid, to which a little of 10-per-cent. solution of trikresol has been added. The pockets should then be left undisturbed for a week, but the gums should be swabbed five or six times daily with tincture of iodine or a mixture of the tinctures of iodine and aconite, and a stimulating astringent mouth-wash used frequently. It is well at the same time to stimulate the lymphatic activity of the gums by massaging them with a soft linen towel previously soaked in the iodine solution. Should the pyorrhœa give rise to neuralgic pains, a 20 per cent. solution of tannic acid in alcohol applied to the gums will frequently remove the pain. The author has obtained good results from the application of the galvanic current directly to the gums for ten minutes daily. This should be done before applying the lactic acid and iodine treatment. If the gums do not heal within ten days the whole process, including the scaling, is repeated, since some irritant must have been left behind. In cases where the disease is aggravated by the presence in the patient of some constitutional malady, suitable treatment must, of course, be instituted to overcome this as far as possible. The author lays stress on the necessity of fully acquainting the patient with the fact that his disease is essentially a chronic one, and that time and careful treatment are necessary before a cure can be obtained.

THE treatment of Post Basic Meningitis by Serum was the subject of a lecture reported in The Hospital on March 20, 1909 (p. 633). An important review of the extension in Paris of this form of serum therapy to epidemic cerebro-spinal meningitis appears from the pen of Dr. J. D. Rolleston in the *British Journal of Children's Diseases*. The epidemic which has prevailed in the French capital has been a somewhat serious one, for some three hundred cases were reported in the first five months of the year. The sera most used have been Flexner's, which Dr. Poynton, at the date of the lecture above alluded to, was unable to obtain (evidently now the difficulty of getting this substance has been overcome), and a serum manufactured by Dopter at the Pasteur Institute. These two sera are those which have given the best results so far. With both of them it is recommended that the injection be made direct into the theca spinalis, not subcutaneously: a quantity of cerebro-spinal fluid about equal in bulk to the serum to be injected should be withdrawn; but ever after a " dry tap," when no cerebro-spinal fluid flows, the serum has often been beneficial. Dopter reports 196 cases treated since the beginning of 1909, with a mortality of only 15.8 per cent.; subtracting cases moribund when first treated, and those dying from other causes, the mortality is but 10.3 per cent. His serum is obtained from horses, and he has now adopted a new method which he believes to produce a serum superior to that which he formerly made. He admits that in three kinds of case his treatment is useless; that is, in cases seen too late, in septicæmic forms, and in cases where cerebral symptoms predominate. Sequelæ and complications are reduced by serum treatment, and recovery is more rapid than under any other treatment.

---

Attention is drawn in the *Journal of Clinical Research* to a laboratory test for Syphilis which is said to be almost infallible, and of far more value than the Wassermann reaction. This consists in obtaining gland juice for films, by gland puncture with a fine aspirating needle, and direct staining for *Spirochæta pallida* in the films thus obtained. A small syringe, such as is used for hypodermic injection, is sterilised by boiling; the skin over the gland is shaved, washed, and prepared with ether, and is then stretched tightly over the gland, whilst the needle is plunged obliquely into the latter. Strong aspiration is employed, and the needle point is moved about inside the gland. The small amount of fluid thus drawn up into the needle is then expelled into a glass slide, and smeared out into a film. Without fixing it may be stained at once by Giemsa's method. When dry the film is ready for examination under an oil immersion lens, and if *Spirochæta pallida* is found, the lesion is syphilitic even if no definite secondaries have appeared. It is claimed by Preis that syphilis can be thus confirmed or excluded in the stage between the appearance of a chancre and the development of a roseola in almost 100 per cent. of cases. Even if the positive evidence alone is thus trustworthy the plan is worth extended trial; and if a negative result is equally good proof of the non-specific nature of a venereal sore, an important advance has certainly been made.

---

Claret and Lyon-Caen, in *Les Bulletins de la Société Médicale des Hopitaux*, reports a case of meningitis due to the typhoid bacillus. The patient, a woman of 23, was admitted into hospital suffering from headache, prostration, and fever, all of which symptoms had lasted for 10 days. On examination she was found to have a furred tongue, dry at the tip and edges, bronchitis, a palpable spleen, and constipation, accompanied by gurgling in the right iliac fossa. Her temperature varied from 103° F. in the morning to 104° F. in the evening. In due course rose spots appeared, and a positive Widal reaction was obtained with a dilution of 1 in 100. Ten days after admission she complained of increased headache and photophobia and a left-sided ptosis was discovered, and in two days' time the other signs of meningitis appeared. Lumbar puncture yielded a fluid free from organisms. The day following this operation was repeated, and a pure culture of bacillus typhosus was obtained from the fluid. Two days later there occurred a hæmorrhage from the bowel. Lumbar puncture was performed 48 hours later, the fluid this time yielding no organisms, though it agglutinated a fresh culture of typhoid bacillus in a dilution of 1 in 20, while at the same time the blood serum agglutinated it in a dilution of 1 in 100. The authors point out the rarity of meningitis due to the typhoid bacillus, and mention the fact that in 12 cases only has the organism been discovered during the patient's life, though it has been found in the sub-arachnoid fluid or pus in some cases after death.

---

In order to avoid the profuse hæmorrhage which may occur unexpectedly in the course of even the most simple operations, Drs. Wolgang Denk and Helleman, of Vienna, have devised a simple and rapid method of estimating the coagulability of the blood prior to operation. A pipette of special construction is used, consisting of a capillary tube some four inches in length, in the course of which are two reservoirs distant from each other about two inches. The total capacity of the instrument is 5 c.c. The tube is filled with blood obtained by pricking the finger, the time at which this is done being carefully noted, and the tube and contents kept as far as possible at body temperature by placing them in water at 98.4° F. between the subsequent manipulations. At short intervals of time drops of blood are allowed to fall on to a piece of filter-paper from the pipette. So long as the blood remains fluid each drop will spread after reaching the paper, but when coagulation commences the drop will cease spreading and a clot will form where the blood touches the paper, while at the same time a filament of fibrin will be seen projecting from the lumen of the tube. The time which elapses between the taking of the blood from the patient and the onset of these phenomena indicates the coagulation time. Normal blood clots in two and a half minutes. In hæmophilia the time is increased to six or seven minutes or more. In cachectic states, and particularly in cancer, coagulation is accelerated and occurs in from one to two minutes. A similar increase in the rapidity of clotting is observed in patients who are liable to post-operative embolism. The authors advise the administration of calcium salts prior to operation in the case of hæmophilics, and of citric acid to those in whom the formation of emboli is to be feared.

---

Some explanation of the abnormal course which accidental affections of the liver may take during pregnancy and menstruation is afforded by an observation of Choosteck (*Wiener Klin. Woch.*) that during the menstrual periods a definite enlargement of the liver occurs which can be demonstrated by percussion. This enlargement occurs quite independently of any morbid condition of the organ, and disappears when the flux ceases, thereby showing that a definite relationship exists between the two. This connection between the liver and the genitalia is comparable to those which are already known to exist between the organ and the intoxications of pregnancy. The author believes that this menstrual hyperæmia of the liver is caused by the products of the internal secretion of the ovaries.

